# Sex-specific effects of Cre expression in Syn1Cre mice

**DOI:** 10.1038/s41598-023-37029-9

**Published:** 2023-06-20

**Authors:** Maarouf Baghdadi, Andrea Mesaros, Martin Purrio, Linda Partridge

**Affiliations:** 1 Max-Planck Institute for Biology of Ageing, Cologne, Germany; 2 Institute of Healthy Ageing, and GEE, UCL, London, UK

**Keywords:** Neuroscience, Behavioural methods, Biological models, Metabolism

## Abstract

The Cre-loxP system has been used to generate cell-type specific mutations in mice, allowing researchers to investigate the underlying biological mechanisms of disease. However, the Cre-recombinase alone can induce phenotypes that confound comparisons among genotypes if the appropriate Cre control is not included. In this study, we characterised behavioural, morphological and metabolic phenotypes of the pan-neuronal Syn1Cre line. We found that these mice possess intact neuromuscular parameters but have reduced exploratory activity and a male-specific increase in anxiety-like behaviour. Moreover, we observed a male-specific deficit in learning and long-term memory of Syn1Cre mice that could be a result of decreased visual acuity. Furthermore, we found that over-expression of human growth hormone (hGH) from Syn1Cre results in a male-specific reduction in body weight and femur length, potentially through decreased hepatic Igf1 expression. However, metabolic characteristics of Syn1Cre mice such as glucose metabolism, energy expenditure and feeding were unaffected by the presence of Syn1Cre. In conclusion, our data demonstrate that Syn1Cre expression has effects on behavioural and morphological traits. This finding highlights the importance of including the Cre control in all comparisons, while the male-specific effects on some phenotypes highlight the importance of including both sexes.

## Introduction

Genetically engineered mice are commonly used to model certain aspects of human disease and to probe specific gene function. One method is the use of transgenic mouse lines in which Cre-recombinase (Cre) is expressed under the control of specific gene promoters, thereby giving the genetic intervention spatial specificity. Cre expressing mice are then mated with mice carrying short target sequences called loxP sites that are targeted to a specific gene. The subsequent progeny from this mating will result in mice where the Cre then excises the section of DNA flanked by loxP sites creating a targeted knockout mouse line. The Cre-loxP system has been developed as a critical experimental tool to achieve spatial control when investigating genotypes of interest in a tissue- or cell-specific manner. Many tissue-specific Cre-driver mouse lines have been generated to target specific cell types within a heterogeneous tissue.

The brain is a complex and heterogeneous tissue, analysis of which has benefited tremendously from the genetic access provided by Cre-drivers to specific brain areas through enriched or restricted gene expression patterns^1^. The brain not only mediates behaviour in an animal but is also a central mediator of peripheral metabolism, largely, but not solely, through the action of the hypothalamus^2^. Therefore, Cre drivers with widespread neuronal expression have been used to understand the role of neuronal function in behaviour and systemic metabolism, such as the Nestin-Cre modal. However, while originally thought to be uniquely driving recombinase expression in neurons, this tool is now widely recognized to affect radial glia as well as their astrocyte progeny^3^. Moreover, the Nestin-Cre tool has a number of pitfalls for conducting metabolism and behavioural research due to Cre expression in peripheral tissues in addition to neurons and glia^4^.

An alternative pan-neuronal Cre-driver to test the roles of neuronal function is Cre driven from the rat-Synapsin I promoter (Syn1Cre), which has been shown to drive transgene expression widely and specifically in neuronal cells in the central nervous system, with the exception of the Purkinje cells in the cerebellum, but not glial cells^5^. In this study we characterised the pan-neuronal Syn1Cre line for applications in behavioural and metabolic studies. We implemented a phenotyping pipeline that assessed a variety of behavioural parameters such as motor performance, exploratory behaviour, anxiety-like behaviour, working memory, and spatial reference memory. We found significant effects of Syn1Cre on exploratory behaviour, and a male specific effect on anxiety-like behaviour and spatial reference memory. We also characterised metabolic parameters and found a significant reduction in body weight of male Syn1Cre mice. However, no significant changes were detected in peripheral metabolic phenotypes such as glucose tolerance, insulin sensitivity, energy expenditure and locomotor activity.

In conclusion, our data demonstrate that Syn1Cre expression has effects on behavioural and morphological traits, and some of these effects were sex-specific. These findings highlight the importance of including the Cre control and both sexes in studies that use the Cre-loxP system.

## Results

### Intact motor learning, neuromuscular strength and non-aversive exploratory behaviour in Syn1Cre mice

Cre endonuclease can have deleterious effects when over-expressed in neurons^6^. Syn1Cre mice express the Cre recombinase across the brain, including in areas that coordinate motor functions^7^. Therefore, we assessed neuromuscular function in Syn1Cre mice using complementary assays measuring different aspects, to establish whether neuronal Cre expression produced any deficits that could confound subsequent behavioural phenotyping.

**Figure 1:**
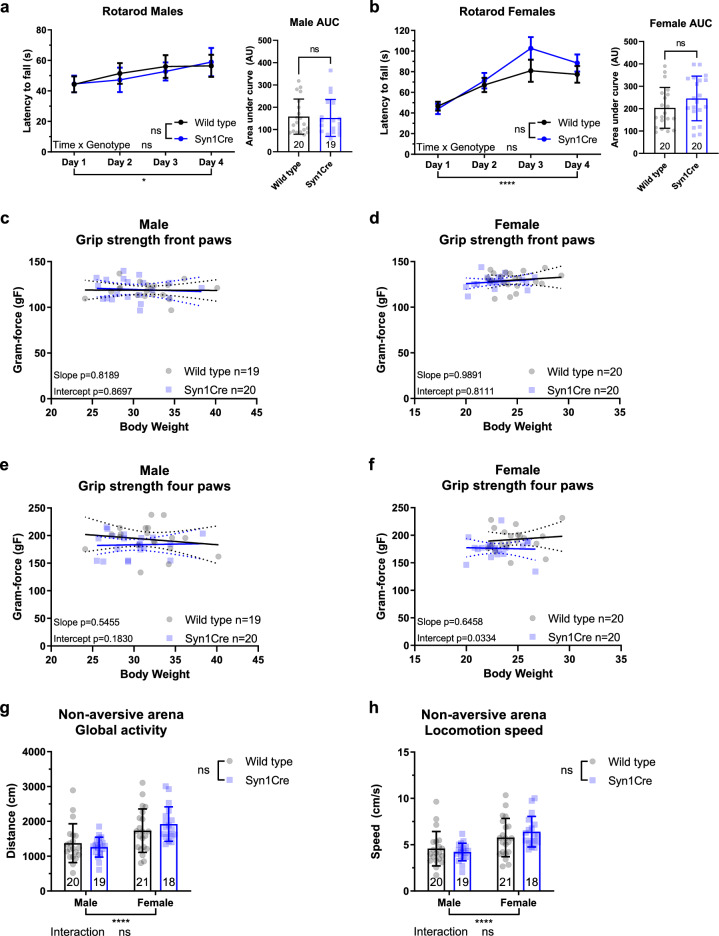
Neuromuscular function is not affected in Syn1Cre mice. Average latency to fall during four days of training in the Rotarod revealed no significant difference in motor learning over time in male (**a**) or female (**b**) Syn1Cre mice. Analysis of body weight adjusted front-paw grip strength revealed no significant difference in strength of male (**c**) or female (**d**) Syn1Cre mice. Analysis of all four paws showed no significant difference in male Syn1Cre mice (**e**), but a significant reduction in body weight-adjusted strength of all four paws in female Syn1Cre mice was detected (**f**). Exploration in a non-aversive arena were not significantly different in Syn1Cre mice (**g**). No significant difference was detected in locomotion speed during exploration of a non-aversive arena in Syn1Cre mice (**h**). Number of animals reported at the bottom of the bars for each condition. All error bars correspond to standard deviation of the mean except for longitudinal rotarod where standard error of the mean is reported. For ANCOVA analysis the 95% confidence interval is plotted. Asterisks denote the following: *P < 0.05, **P < 0.01, ***P < 0.001, ****P < 0.0001. For detailed statistical values see Supplementary Table S1.

We performed a rotarod experiment to assess if 14-week-old Syn1Cre mice showed loss of motor coordination or balance^8^. We measured the average latency of a mouse to fall from an accelerating rotating rod across four training days. This assay not only evaluated motor coordination and balance as the mouse adjusted to the increasing speed of rotation to avoid falling, but also determined the motor learning capacity by measuring the increase in performance across training days. The increase in average latency to fall across time was analysed by repeated measures ANOVA, and revealed no motor learning deficits in either male (Fig. 1a) or female (Fig. 1b) Syn1Cre mice. Moreover, no deficit in motor performance was detected by analysing the rotarod area under the curve (AUC) of either male (Fig. 1a) or female (Fig. 1b) Syn1Cre mice.

For the evaluation of neuromuscular strength, we performed a grip strength assay for only front paws or all four paws of 15-week-old Syn1Cre mice. Given the influence of body weight on the results, we used analysis of covariance (ANCOVA) using body weight as a covariate to assess if grip strength was affected^9^. Front paw grip strength of male (Fig. 1c) and female (Fig. 1d) Syn1Cre mice were unaffected. Four paw grip strength of male (Fig. 1e) Syn1Cre mice also did not show any significant difference. However, a small but significant reduction in female (Fig. 1f) four paw grip strength was detected.

We measured locomotor and exploratory activity in free behaving 16-week-old Syn1Cre mice in a non-aversive arena for five minutes. This experiment allowed determination of activity without the presence of any anxiety-provoking stimulus. We observed no significant difference in the distance travelled (Fig. 1g) or locomotion speed (Fig. 1h) of male or female Syn1Cre mice. This data suggests that, in the absence of anxiety-provoking stimuli, there is no deficit in the motivation and capacity of Syn1Cre mice to explore an area.

Taken together, our data suggests that neuromuscular function and activity are unaffected in Syn1Cre mice.

### Syn1Cre mice show reduced exploration and a male-specific increase in anxiety-like behaviour

**Figure 2:**
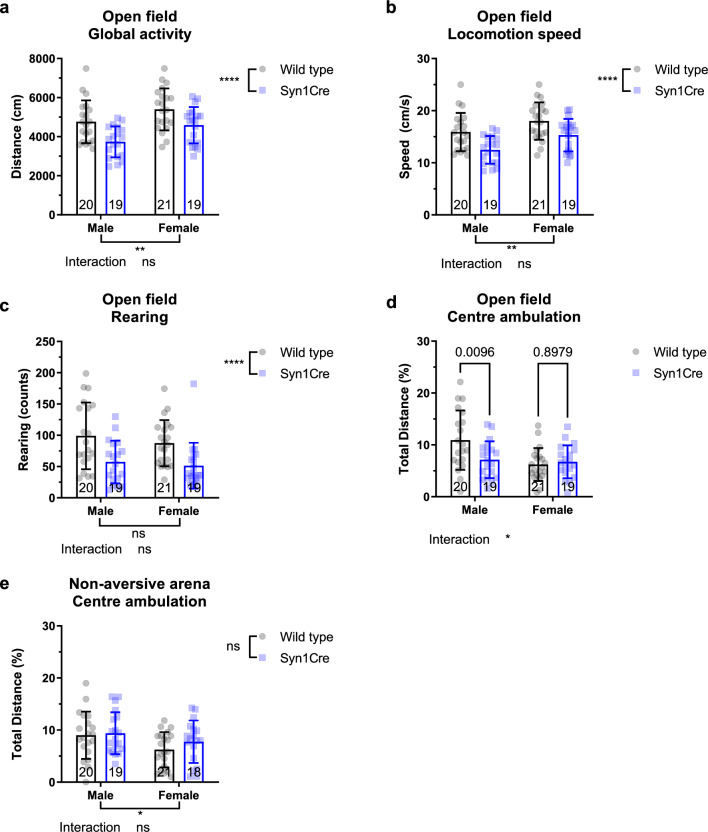
Exploratory and anxiety-like behaviour in Syn1Cre mice. (**a**) Locomotor activity and (**b**) locomotor speed in the open field arena were significantly reduced in Syn1Cre mice. (**c**) Rearing behaviour was significantly reduced in Syn1Cre mice. (**d**) Male but not female Syn1Cre mice showed reduced centre occupancy. (**e**) Centre ambulation normalised to total distance measured in the non-aversive arena revealed no significant difference in Syn1Cre mice. Number of animals reported at the bottom of the bars. All error bars correspond to standard deviation of the mean. Interaction between genotype and sex was analysed by two-way ANOVA, followed by Sidaks post hoc test. Asterisks denote the following: *P < 0.05, **P < 0.01, ***P < 0.001, ****P < 0.0001. For detailed statistical values see Supplementary Table S1.

In the absence of any baseline deficits in locomotor capabilities in Syn1Cre mice, we assessed exploration and anxiety-like behaviour using an open field assay^10^. 12-week-old mice were placed in an anxiety provoking open field arena with a highly illuminated centre (~ 220 lx) and clear walls for five minutes. Total distance covered was significantly reduced in both female and male Syn1Cre mice (Fig. 2a). Moreover, average locomotion speed during exploration of the open field was also significantly lower in Syn1Cre mice (Fig. 2b). Rearing counts were assessed as another measure of exploratory behaviour. Syn1Cre mice had a significantly lower number of rearing counts (Fig. 2c). These data suggest that the presence of anxiogenic stimuli, such as bright lights and clear walls, lead to reduced motivation of Syn1Cre mice to explore an arena compared to the non-aversive arena (Fig. 1g,h).

To assess anxiety-like behaviour in Syn1Cre mice, we measured the distance travelled in the centre of the highly illuminated arena^10^. However, given the difference in total distance travelled in the open field, we normalised distance travelled in the centre by total distance travelled in five minutes. Interestingly, we detected a significant sex-specific decrease in centre occupancy (two-way ANOVA, sex*genotype interaction P = 0.021; F (1,75) = 5.553, Fig. 2d). Male Syn1Cre mice had significantly lower distance travelled in the centre of the arena suggesting that Syn1Cre expression leads to a male-specific increase in anxiety (Fig. 2d). To confirm that the reduction in centre occupancy was due to anxiety-like behaviour, we measured centre occupancy in the non-aversive arena where mice were not exposed to the anxiogenic stimulus. We found no significant difference in male or female Syn1Cre distance travelled in the centre of the darker arena (Fig. 2e), suggesting that the phenotype observed in Syn1Cre males was due to anxiety-like behaviour.

### Learning and long-term spatial reference memory is impaired in Syn1Cre mice

**Figure 3:**
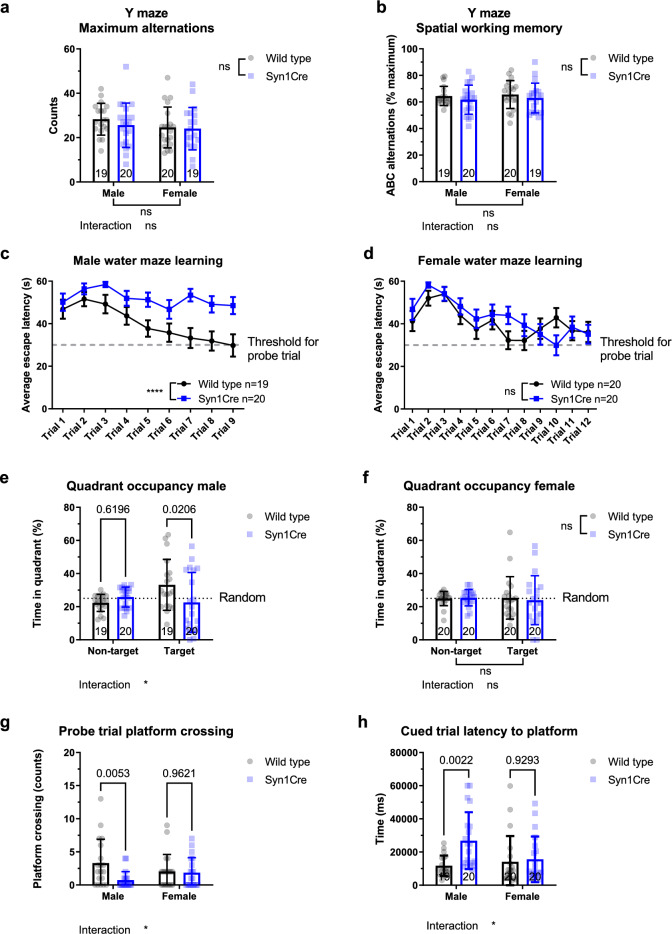
Spatial reference memory and working memory of Syn1Cre mice. (**a**) Number of alternations during the spontaneous Y-maze assay revealed no significant difference in Syn1Cre mice. (**b**) Number of correct A > B > C alternations as a percentage of total alternations did not show any significant difference in Syn1Cre mice. (**c**) Assessment of escape latency across nine trials of water maze training showed significant reduction in learning of male Syn1Cre mice. (**d**) Assessment of escape latency across 12 trials of water maze training showed no significant difference in female Syn1Cre mice (female wild type n = 19 and Syn1Cre n = 20). (**e**) Probe trial quadrant occupancy revealed a significant reduction in target quadrant occupancy in male Syn1Cre mice compared to wild type littermates (male wild type n = 19 and Syn1Cre n = 20). (**f**) Probe trial quadrant occupancy revealed no memory in female wild type or Syn1Cre mice (female wild type n = 19 and Syn1Cre n = 20). (**g**) Platform crossings during probe trial revealed presence of long-term memory exclusively in male wild type mice. (**h**) Latency to escape in the cued water maze revealed significant sex-specific increase of time in male Syn1Cre mice. Number of animals reported at the bottom of the bars where possible, otherwise in the figure legend. All error bars correspond to standard deviation of the mean except water maze training where standard error of the mean was plotted. Asterisks denote the following: *P < 0.05, **P < 0.01, ***P < 0.001, ****P < 0.0001. For detailed statistical values see Supplementary Table S1.

We used the spontaneous alternation version of the Y-maze to evaluate spatial working memory in 13-week-old mice, as this task does not rely on swimming ability and visual acuity but also the vestibular system^11,12^. We measured total locomotor activity and locomotor speed (Supplementary Fig. S1) in the Y-maze and could not detect any significant difference in Syn1Cre mice. When we measured the number of total arm entries in Syn1Cre mice, we could not detect any significant difference, suggesting that both genotypes were equally likely to explore the maze (Fig. 3a). When we measured the number of alternations where the mouse favoured novel arms over previously visited arms (so called ABC alternations), we found no significant deficits in male or female Syn1Cre mice (Fig. 3b). These data suggest that short-term memory formation is not affected in Syn1Cre mice.

An important function of the brain is hippocampus-dependent spatial learning and memory, which can be assessed using a water maze^13,14^. The water maze, a test of spatial reference memory, allows assessment of both learning and memory was performed in 17-week-old Syn1Cre mice. To assess the capacity of Syn1Cre mice to learn, mice were placed in a water arena with a hidden platform and the latency to escape the water, by using spatial cues to find the hidden platform, was recorded. Male Syn1Cre mice were trained in the water maze task for nine trials until wild type mice reached the threshold indicating adequate learning (< 30 s). However, Syn1Cre males could not reliably find the hidden platform in the same amount of time as wild type males after equal training time, indicating a learning deficit in the Syn1Cre males (Fig. 3c). Female Syn1Cre mice learned the location of the hidden platform as well as did the wild type littermates but took 12 trials to reach the learning threshold (< 30 s) (Fig. 3d).

To address long term memory, a probe trial was administered 24 h after the last training session, when the hidden platform was removed and the animal was allowed to search for the platform for 60 s. During the probe trial, we detected a significant reduction in total swim path (Supplementary Fig. S1) and locomotion speed (Supplementary Fig. S1) of Syn1Cre mice, similar to the results we observed in the open field task (Fig. 2a,b). Long-term memory formation was measured by occupancy of the target quadrant previously containing the hidden platform and the number of platform crossings. Male Syn1Cre mice had a significantly reduced target quadrant occupancy (Fig. 3e). Moreover, the number of platform crossings was significantly lower in male Syn1Cre mice compared to wild type littermates (Fig. 3g). These data suggests that, unlike wild type mice, male Syn1Cre mice failed to form a memory of the hidden platform location in nine trials of training. Although we saw significant learning in female wild type and Syn1Cre mice (Fig. 3d), we could not detect evidence of memory formation 24 h later in either quadrant occupancy (Fig. 3f) or in platform crossing (Fig. 3g).

Additionally, we administered a visually cued variant of the water maze to assess the visual ability of Syn1Cre mice, as the water maze task depends on intact visual acuity. The cued water maze was administered 24 h after the probe trial. The platform location was changed, and the spatial cues were covered using a curtain around the maze to motivate the animals to use the new proximal cue on the platform to escape. Three training trials were administered from different start locations and the average of the three trials was used to assess visual acuity. This task involves the same motor movements (swimming), motivation and reinforcement (escape from water) as the hidden water maze, however, it differed in that the mice were not required to learn the position of the platform in relation to any distal room cues. We detected no significant difference in swim path (Supplementary Fig. S1) but a significant reduction in swim speed (Supplementary Fig. S1) of Syn1Cre mice in the cued variant of the water maze. The reduction in swim speed may suggest impaired swimming performance in Syn1Cre mice. The latency to the escape platform in the cued water maze was measured and we detected a significant, sex-specific difference (two-way ANOVA, sex*genotype interaction P = 0.033; F (1,75) = 4.733, Fig. 3h). Male Syn1Cre mice took significantly longer to escape the water maze even when a proximal visual cue was placed on the platform (Fig. 3h). This suggests that swimming performance and visual deficits can be some of the factors contributing to the phenotypes of male Syn1Cre mice preventing them from using proximal or distal cues to locate the platform in the same number of trials or time as the male wild type controls (Fig. 3c). Therefore, the impairment in learning and memory deficits we observed in male Syn1Cre mice (Fig. 3c,e) may in fact have been due to deficits in swimming ability and visual acuity. Moreover, the data suggests that the memory deficit in female mice may be due to insufficient training trials as they possessed similar motivation, swimming ability and visual acuity to escape during the cued and hidden platform version of the water maze.

### Neuronal Syn1Cre expression leads to increased hypothalamic hGH transcript levels and a male-specific decrease in bone size

**Figure 4:**
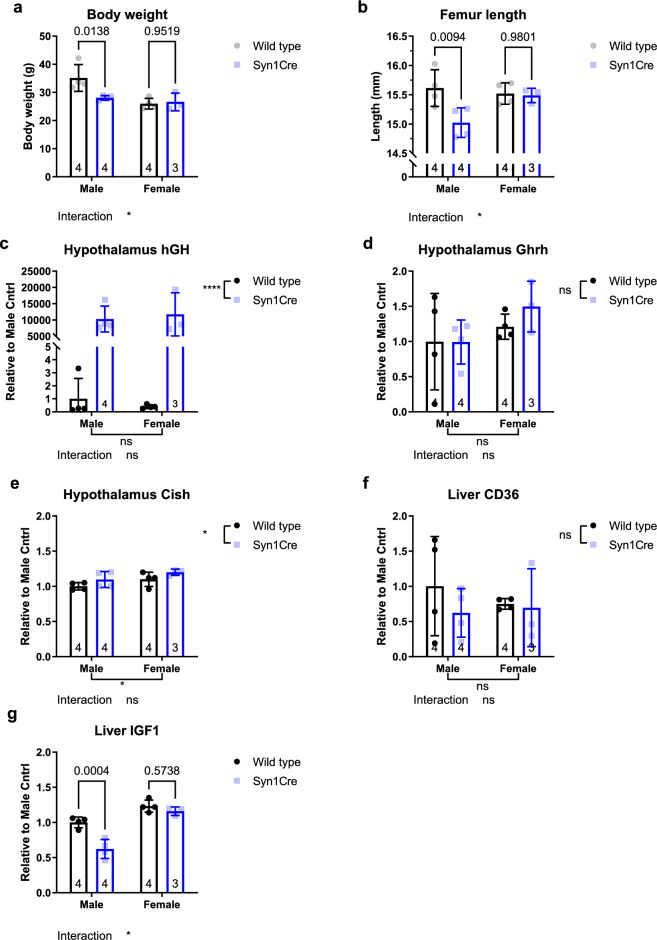
Cre transgene triggers increased hGH expression leading to a sex-specific reduction in body size of male Syn1Cre mice. (**a**) Two-way ANOVA analysis of Syn1Cre body weight revealed a sex-specific significant reduction in Syn1Cre males. (**b**) Two-way ANOVA analysis of femur length revealed a sex-specific reduction in male Syn1Cre femur size. (**c**) Measurement of hGH transcript levels using quantitative real-time PCR (qPCR) in the hypothalamus revealed significant increase in hGH expression in Syn1Cre mice. (**d**) No significant difference in Ghrh levels in hypothalamus extracts between Syn1Cre mice and wild type littermates. (**e**) Significant increase in Cish transcript levels in the hypothalamus of Syn1Cre mice. (**g**) Measurement of CD36 transcripts by qPCR in liver tissue revealed no significant difference between Syn1Cre mice and wild type littermates. (**f**) Two-way ANOVA of Igf1 transcript levels in Syn1Cre mice revealed a sex-specific reduction of hepatic Igf1 expression in male Syn1Cre mice. Number of animals reported at the bottom of the bars. All error bars correspond to standard deviation of the mean. Asterisks denote the following: *P < 0.05, **P < 0.01, ***P < 0.001, ****P < 0.0001. For detailed statistical values see Supplementary Table S1.

Syn1Cre mice showed a male-specific reduction in body weight (two-way ANOVA, sex*genotype interaction P = 0.032; F (1,11) = 6.048, Fig. 4a). Moreover, the difference in body weight was not due to a change in body composition as body fat as a percentage of body weight was not significantly different in male or female Syn1Cre mice compared to wild type controls (Supplementary Fig. S3). To investigate whether the reduction in weight was due to reduced body size, we analysed the femur length of the same animals. Indeed, we found a consistent male-specific reduction in femur length of Syn1Cre mice (two-way ANOVA, sex*genotype interaction P = 0.045; F (1,11) = 5.127, Fig. 4b). Furthermore, a previous study reported similar findings of a reduction in body weight due to Cre expression under a neuronal promoter in male Nestin-Cre mice^15^, which was attributed to disrupted growth hormone (GH) signalling, as a human growth hormone (hGH) minigene was inserted downstream of the Cre recombinase to achieve higher expression of the Cre transgene^16^.

The Syn1Cre mouse line was also generated by placing the hGH gene 3’ of the Cre transgene under the rat-Synapsin1 promoter^5^. Therefore, we measured hGH transcript levels in hypothalamus of the Syn1Cre mice by quantitative real-time PCR (Q-RT-PCR), and found a significant increase (Fig. 4c). To test whether the hGH signal we observed was due to the hGH from Cre and not the endogenous mouse growth hormone, we used a forward annealing primer to the Cre fragment and a reverse primer annealing to the hGH minigene. We found a similar fold change between the Ct value of the Cre transgene and the B2M housekeeping gene (Supplementary Fig. S2), and the Cre-hGH transgene and B2M (Supplementary Fig. S2). The hGH results were not due to primer binding to the endogenous mouse genome as we either could not detect or only detected a signal at 34 cycles in wild type mouse hypothalamus relative to detection at 18 cycles of the B2M housekeeping gene. In summary, Syn1Cre mice had increased expression of exogenous growth hormone transcript in the hypothalamus due to the Cre-hGH transgene, which might indicate disrupted GH signalling.

To address whether altered growth hormone (GH) signalling could explain the decrease in body weight and size observed in male Syn1Cre mice, we performed transcriptional assays to study downstream GH activity. GH secretion from the pituitary is regulated by hypothalamic GH releasing hormone (*Ghrh*), which promotes GH secretion, and somatostatin, which inhibits release of GH^17^. Previous reports have found that expression of *hGH* in the hypothalamus of mice can lead to reduction of hypothalamic *Ghrh* leading to GH deficiency^18^. Surprisingly, when we tested the levels of *Ghrh* in the hypothalamus, we detected no significant difference in Syn1Cre mice (Fig. 4d). Next, we asked whether the expression of hGH led to an activation of the STAT5 pathway, a central mediator of the effect of GH signalling on body size^17^. We measured the expression of cytokine inducible SH2-containing protein (*Cish*), which is activated after phosphorylation of STAT5^15^. We observed a small but significant increase in expression of *Cish* in Syn1Cre mice (1.149 ± 0.098-fold change) (Fig. 4e), suggesting increased GH signalling in the hypothalamus^17^. To assess if there was a similar effect on GH signalling systemically, we measured CD36 transcripts in the liver of Syn1Cre mice due to the role of STAT5 in hepatic lipid metabolism^19^. We could not detect a significant difference in hepatic transcript levels of CD36 in Syn1Cre mice (Fig. 4f). However, we detected a male-specific reduction of *Igf1* expression in Syn1Cre mice (two-way ANOVA, sex*genotype interaction P = 0.011; F (1,11) = 9.193, Fig. 4g). Hepatic IGF1 is downstream of GH signalling^17^ and is a primary mediator of body size^20^, and its reduction may have been a contributing factor to the reduction in male Syn1Cre bone size and body weight (Fig. 4a,b). Interestingly, a previous report on Nestin-Cre mice saw a similar reduction in male hepatic *Igf1* transcripts^15^. We found that male Syn1Cre mice have reduced expression of hepatic *Igf1* through a STAT5 independent mechanism, suggesting a negative feedback loop is triggered indirectly in male, but not female, Syn1Cre mice.

### Intact systemic metabolic parameters in Syn1Cre mice

The hypothalamus is a central mediator for peripheral insulin sensitivity and glucose metabolism^2^. We therefore assessed whether Cre recombinase expression in the brain led to any effects on peripheral metabolism in Syn1Cre mice.

**Figure 5:**
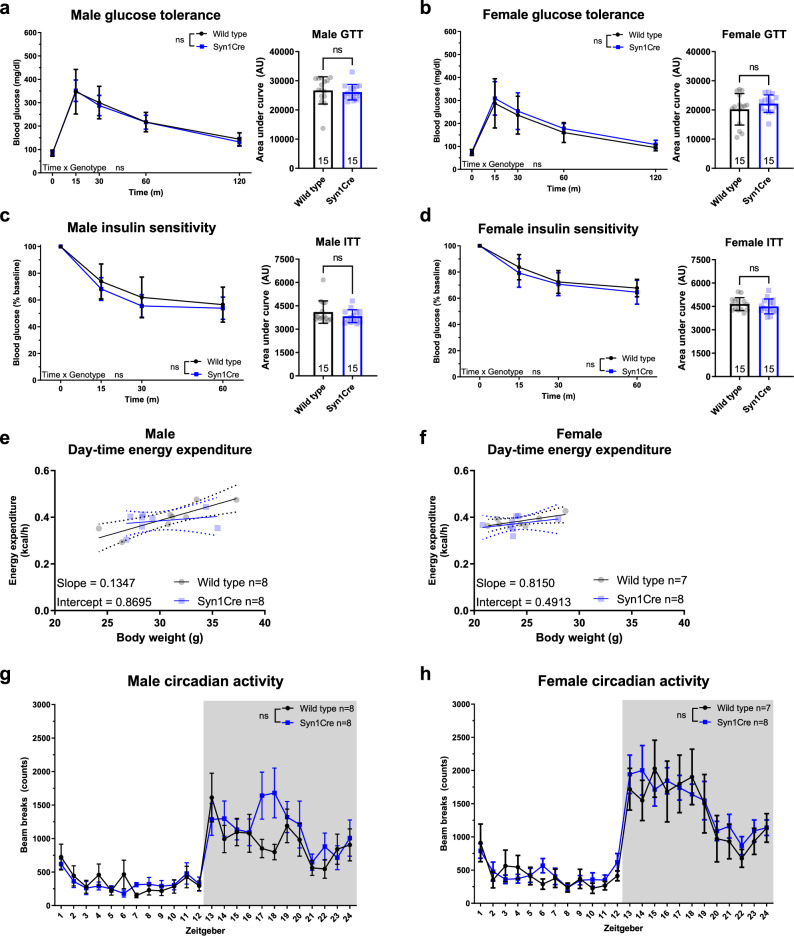
Syn1Cre expression has no effect on glucose homeostasis or energy expenditure. Glucose tolerance test (GTT) on male Syn1Cre mice (**a**) and female Syn1Cre mice (**b**) did not detect any significant difference relative to their respective wild type littermates. Insulin sensitivity test (ITT) on male Syn1Cre mice (**c**) and female Syn1Cre mice (**d**) did not detect any significant difference relative to their wild type littermates. ANCOVA on day-time energy expenditure of Syn1Cre mice showed no significant difference between male (**e**) or female (**f**) Syn1Cre and their respective wild type littermates (male wild type and Syn1Cre n = 8, female wild type n = 7 and Syn1Cre n = 9). Locomotor activity of single-housed male (**g**) and female (**h**) Syn1Cre mice plotted against zeitgeber revealed no significant difference between Syn1Cre mice and wild type littermates. Number of animals reported at the bottom of the bars. All error bars correspond to standard deviation of the mean. For ANCOVA analysis the 95% confidence interval is plotted. Asterisks denote the following: *P < 0.05, **P < 0.01, ***P < 0.001, ****P < 0.0001. For detailed statistical values see Supplementary Table S1.

We assessed glucose tolerance by administration of a glucose tolerance test (GTT) after an overnight fast. This gives insight into pancreatic beta cell function as well as the capability of an animal to suppress hepatic gluconeogenesis in response to a body-weight-adjusted glucose challenge. Area under the curve (AUC) analysis of GTT revealed no significant difference in the glucose sensitivity of male (Fig. 5a) or female (Fig. 5b) Syn1Cre mice compared to the respective wild type controls. Insulin sensitivity is a cornerstone of metabolic health, since it reflects the ability for insulin to activate peripheral tissue absorption of blood glucose. To assess systemic insulin sensitivity, we administered an insulin tolerance test (ITT) which measures changes in blood glucose levels in response to a body weight-adjusted bolus of insulin injected intraperitoneally. AUC analysis of ITT revealed no significant difference in insulin sensitivity of male (Fig. 5c) or female (Fig. 5d) Syn1Cre mice relative to their wild type controls.

The hypothalamus in the brain is a mediator of energy expenditure, circadian rhythm and locomotor activity in an animal^21^. Therefore, we assessed energy expenditure and circadian rhythm, given the effects we observed in the hypothalamus. We placed single-housed mice in metabolic cages that allowed measurement of indirect calorimetry. We used ANCOVA analysis using body weight as a covariate to analyse if there were differences in energy expenditure between Syn1Cre mice and their wild type littermates. We did not detect any significant differences in day-time (Fig. 5e) or night-time (Supplementary Fig. S3) energy expenditure in male or female (Fig. 5f and Supplementary Fig. S3) Syn1Cre mice. We used beam breaks during the animal’s time in the metabolic cages to infer average home cage activity levels across a 24-h period. We found no significant difference in the amount or phase of activity in male (Fig. 5g) or female (Fig. 5h) Syn1Cre mice. Finally, we used respiratory exchange ratio (RER) measurements in the metabolic cages to determine the type of fuel consumption during different phases of the day in Syn1Cre mice. Consistent with our locomotor activity and energy expenditure findings, we did not detect any significant difference of RER in male or female (Supplementary Fig. S3) Syn1Cre mice.

All together we found no significant effect of neuronal Cre recombinase expression on peripheral metabolism, validating the use of this tool to study the neuronal control of glucose metabolism.

## Discussion

In this study we report behavioural and metabolic phenotyping of Syn1Cre mice. We focused on phenotypes relevant for metabolic and neuropsychiatric disorders, as those are the primary applications for pan-neuronal genetic tools. Sex is increasingly recognised as an important influence on the brain in health^22^, disease^23^ and metabolism^24^. Therefore, we characterised both male and female Syn1Cre mice. Interestingly, we found sex-specific effects of Syn1Cre expression on behaviour and body size.

Many of the previous reports on the neuronal control of metabolism have used Nestin-Cre mice. However, given the recent evidence of the metabolic pitfalls such as a reduction in lean mass, body size and insulin insensitivity, alternative models are being sought^4^. Some of these effects come from altered GH signalling (body size) while others imply off-target effects of the Cre expression in other cell types such as the pancreas^25^. An alternative and specific pan-neuronal gene driver is required. An alternative genetic tool is the Cre driven by rat Synapsin-1 promoter, as the only off-target effect reported has been expression in testes. The effects of Syn1Cre expression in the testes leading to germline deletion in the offspring can be easily avoided with a breeding strategy mating Syn1Cre females with floxed males. According to the mouse genome database, more than 170 studies have used Syn1Cre mice^26^, but a study of the effect of Syn1Cre expression on basic phenotypes in males and females is missing. Therefore, we performed extensive characterisation of behavioural and metabolic parameters in young (12- to 18-week-old) Syn1Cre mice.

We did not observe any significant effects on motor learning, neuromuscular strength, or locomotor activity in male or female Syn1Cre mice. However, when mice were placed in the aversive open field, Syn1Cre mice exhibited reduced exploratory drive. In addition, we detected a male-specific increase in anxiety-like behaviour in the aversive open field, a trait that was previously shown to be modified by sex^24^. Centre occupancy in a non-aversive arena further confirmed that the observed phenotype was mediated by the anxiogenic illumination of the open field. Interestingly, previous reports in Nestin-Cre mice also found an increase in anxiety-like phenotype in male mice^27^, suggesting a shared cause in these two genetic drivers due to Cre expression in neurons. When we assessed the learning capability of Syn1Cre mice, again we detected a significant deficit in the ability of male Syn1Cre mice to use distal spatial cues to locate the hidden platform in a water maze, unlike wild type males and both female groups. The probe trial further confirmed the deficit in memory formation in male Syn1Cre mice. Surprisingly, both female groups also did not perform in the probe trial. However, given that wild type females did not show enriched target quadrant occupancy, we could not conclude if female Syn1Cre mice exhibited deficits in memory formation or simply the number of training trials was insufficient. We also administered cued trials in the water maze to test the motivation and visual acuity in Syn1Cre mice. We detected a deficit in male Syn1Cre mice, as they were the only group to have a significantly increased latency to the platform due to slower swim speed when a proximal cue was added. This data suggests that male Syn1Cre mice are not appropriate for use in assays that require intact visual acuity or swimming ability. Data from the cued trial confirmed that the females did not perform the task due to technical reasons and not due to deficits in motivation or visual acuity. A potential reason for these visual deficits is the presence of microphthalmia and anophthalmia in C57BL/6 mice (~ 20% of all females and ~ 2% of male mice)^28^. It is important to note that while the cued water maze is a tool for evaluating visual and motivational capacity of mice to perform the water maze, it should be complemented with other assessment methods to obtain a comprehensive understanding of mice's visual capabilities. However, our data from male Syn1Cre mice suggests the presence of an interaction between the strain and the genotype leading to increased visual defects in male mice. Data from the Y-maze, a test where the vestibular system can also aid the mice to explore the maze^13,14^, suggested that short-term memory formation is still functional in Syn1Cre mice.

Similar to results from Nestin-Cre mice^15^, there was a significant reduction in the body weight of male Syn1Cre mice. Moreover, we demonstrated that, at least in Syn1Cre mice, this effect was male-specific. Furthermore, we confirmed that bone size is a contributor to the sex-specific reduction in body weight we observed. Consistent with the Nestin-Cre study, we detected over-expression of Cre-hGH transcripts due to the design of the Syn1Cre construct^5^. Studies on the effects of Nestin-Cre expression on GH levels are not conclusive, some studies reported an increase in pituitary GH levels^15^ while others detected a significant reduction^29^. Therefore, we decided to look at the downstream consequences of altered GH signalling. Although we found high hGH transcripts in the hypothalamus of Syn1Cre mice and slight upregulation of *Cish* which is downstream of STAT5 signalling, we could not detect STAT5 activation in the liver, unlike the Nestin-Cre model^15^. One potential explanation for this discrepancy could be hGH expression in the multiple peripheral organs that have been reported to express Nestin during development and postnatally^4^. In contrast, Syn1Cre expression is confined to mature neurons and testes, mitigating the potential confounds in this genetic tool^4^. Although we observed a similar over-expression of exogenous hGH in the hypothalamus in Syn1Cre mice, the downstream consequences on STAT5 signalling in the brain and the liver were modest relative to Nestin-Cre mice. We did not observe a reduction in *Ghrh* expression and only a small increase in *Cish* expression, unlike the ~ twofold increase observed in hypothalamus of Nestin-Cre mice. Despite the lack of apparent changes in GH signalling in the hypothalamus of Syn1Cre mice, we did observe a significant male-specific reduction in GH signalling in the liver, as *Igf1* expression was 60% of wild type controls. A similar effect was observed in Nestin-Cre mice where *Igf1* levels were 51% of wild type control males^15^. This was attributed to reduced STAT5 activity in the livers of Nestin-Cre mice due to reduced mGH secretion from the pituitary^15^. However, unlike reports in Nestin-Cre mice that reported a fivefold increase in *CD36* expression due to reduced STAT5 activity, no effect was observed in Syn1Cre mice on downstream STAT5 signalling. Therefore, the effects on *Igf1* expression are independent from STAT5 signalling in Syn1Cre mice. The reduction in *Igf1* levels were consistent with the effects we observed on bone size, as the liver is the primary source of endocrine IGF1 and the contribution of hepatic IGF1 signalling on bone growth is magnified relative to other sources of IGF1^20^. Surprisingly, even though we observed a significant sex-specific reduction in *Igf1* expression in male Syn1Cre mice, we could not detect any significant effect on peripheral metabolism in young and old (16-month-old) mice^30^, namely body composition, glucose tolerance, and insulin sensitivity in male or female Syn1Cre mice. One potential reason could be a compensatory decrease in IGF-binding proteins (IGFBP) that restore normal physiological pathway activity. It is still unclear why Syn1Cre expression leads to effects on *Igf1* expression in males and not females. However, manipulations of the IGF1 pathway result in notoriously sex-specific effects even though IGF1 levels and GH levels are similarly affected in both sexes^31^.

In summary, our study describes for the first time a comprehensive characterization of the frequently used Syn1Cre mouse line in neurobiology. Importantly, our data indicate that indirect effects from the techniques used to generate the model can confound certain behavioural tests in a sex-specific manner, but not general metabolic assays. Moreover, our study highlights the need of including both male and female mice in future studies as well as the use of appropriate experimental control groups to delineate the effects of the Cre transgene.

## Methods

### Mouse generation and husbandry

Cre recombinase under the rat-Synapsin 1 promoter (*Syn1Cre* mice) have been previously generated and deposited in Jackson laboratory under JAX stock #003966^5^. Briefly, The Syn1 Cre transgene was constructed using the SalI–XhoI 4.4-kb fragment of pBL4.3Syn–CAT, containing the rat Synapsin I promoter and 100 bp of 5′ untranslated CAT sequence. This was placed immediately 5′ of a modified bacteriophage P1 Cre recombinase gene that included translational consensus and nuclear localization signals. The human growth hormone gene was placed 3′ of Cre to provide transcription termination and polyadenylation signals to increase transcriptional efficiency. The transgene was isolated from plasmid sequence by NotI digestion and injected into pronuclei of fertilized C57BL/6 × CBA (F2) zygotes. Founder line 671 animals were bred to wildtype C57BL/6NHsd mice. The transgene integrated into chromosome 6, as detected by targeted locus amplification (TLA) (Jackson Laboratory JAX stock #003966). The mice were maintained at the Jackson Laboratory in the C57BL/6J strain. We backcrossed these animals for at least six generations in the C57BL/6N before generating the different mouse cohorts used for molecular biology, behavioural and metabolic phenotyping.

All mice were randomly allocated to groups of four to five same-sex individuals under specific pathogen-free conditions in individually ventilated cages (Techniplast UK Ltd, Kettering, Northamptonshire, UK) in a controlled temperature and humidity environment with 12-h light/dark cycle (lights on from 06:00 to 18:00) and provided ad libitum access to food [ssniff® R/M-H phytoestrogen-poor (9% fat, 34% protein, 57% carbohydrate) ssniff Spezialdiäten GmbH, Soest, Germany] and water. Sentinel mice in the animal room were regularly checked for mouse pathogens according to Federation of the European Laboratory Animal Science Association (FELASA) recommendations^32^.

#### Mouse ethics declaration

Mouse experiments were approved under the ethical permission request 84-02.04.2014.A424 by the North Rhine-Westphalia State Agency for Nature, Environment and Consumer Protection (Landesamt fur Natur, Umwelt und Verbraucherschutz Nordrhein-Westfalen), Germany. Experiments were performed in accordance with the recommendations and guidelines of the state agency and FELASA as well as the ARRIVE guidelines (https://arriveguidelines.org).

#### Mouse genotyping

Transgenic mice were identified by PCR genotyping using DNA extracted from ear clip biopsy and amplified using GoTaq® G2 DNA Polymerase, and tail clips were taken from mice at death for genotype confirmation. Primers used to genotype as well as expected size of amplicon of Syn1Cre line are listed in Supplementary Table S2.

#### Mouse tissue collection

Mice were euthanized at three months of age by transcardial perfusion with PBS + EDTA, after general anaesthesia with a cocktail of Ketamine (120 mg/kg) and Xylazine (10 mg/kg) with supplementary Isoflurane (5%) until no reflex response was observed. Blood was collected by cardiac puncture in tubes with EDTA, plasma-EDTA was isolated by centrifugation at 1,000 g for at least 10 min at 4 °C before aliquoting and storage at − 80 °C. Mice were rapidly decapitated, then the skull and body were dissected by two people simultaneously to minimise tissue deterioration. The brain was removed from the skull and different brain regions were isolated and snap-frozen in liquid nitrogen. The body of the animal was dissected and organs were snap frozen in liquid nitrogen (Supplementary Fig. S4).

### Mouse Behaviour Phenotyping

All behavioural tests were conducted in dedicated behavioural testing rooms starting at 08:00 during the light phase of the light/dark cycle. All mice were handled for multiple subsequent days prior to the start of the phenotyping pipeline. Order of the phenotyping pipeline was performed from the least to most stressful assay (Supplementary Fig. S4). Mice were three months old at the start of the phenotyping pipeline. Mice were randomised and experimenters were blinded to genotype and all experiments were performed by a single, male scientist. Mice were habituated to the room for at least 45 min before behavioural experiments started. Mice were not assayed on days when their home cages were changed. Experimental apparatus was cleaned with bacillol foam (Bacillol AF) between tests on different mice.

#### High and low stress open field

For the high stress open field experiment, mice were left to explore a square arena surrounded by clear walls (50 × 50 × 40 cm) for 5 min for Syn1Cre mice. Different parameters such as total distance travelled, movement speed and time spent in the central area were recorded. Anxiety was assessed by quantifying time or distance spent in the centre as a percent of total distance in the arena. The test chamber was illuminated to 210–220 lx. Data collection was automatic using the TSE-Phenomaster software.

For the low stress non-aversive arena, mice were left to explore a square area surrounded by opaque white acrylic walls (50 × 50 × 40 cm) for 5 min for Syn1Cre mice. The test chamber was illuminated to 20–30 lx. Data collection was automatic using the TSE VideoMot software.

#### Rotarod

We used the rotarod test to test motor coordination and balance. The rotarod uses an accelerating rod^21^, to measure a mouse’s ability to maintain its balance on the rod. The rotation of the rotarod was accelerated from 5 to 40 rpm over a 300 s period. Each mouse was placed twice onto the rod per day, and latency to fall off the rod was measured and averaged, therefore, standard error of the mean was plotted on the graphs depicting rotarod results. The inter-trial interval was approximately one hour. Mice were tested for four consecutive days to assess motor learning.

#### Grip strength

We measured grip strength by gently pulling a mouse after it had gripped a bar assembly until the grip was released. The average gram-force required to release the animal’s grip was measured across five trials (separately). A triangle-shaped bar and a grid-shaped platform were used to measure front paw and four paw strength respectively. Grip strength of front paw and all four paws were measured on different days to prevent any confound of fatigue.

#### Y-maze

Mice were placed in a Y-shaped maze with three opaque black plastic arms at a 120° angle from each other. Mice started at the centre of the maze, then were allowed to freely explore the three arms. Over the course of multiple arm entries, the tendency of a healthy mouse is to enter a less recently visited arm, or to be attracted to the novel arm. The number of arm entries and the number of ABC alternations (where A, B and C are the three different arms) are recorded in order to calculate the percentage of alternation. If mice score around 50% ABC alternations, or chance level, that means mice were equally likely to enter the previously visited arm or the novel arm suggesting that there are deficits to working memory formation. An entry occurs when all four limbs are within the arm for longer than 500 ms. Each mouse was placed in the maze for eight minutes.

#### Hidden platform and visual cued water maze

The hidden platform variation of the water maze was used to examine spatial learning ability and long-term reference memory. We adapted the water maze protocol from a previous report^11^. The water maze consists of a round basin with a diameter of 1.2 m filled with water made opaque by adding white, non-toxic paint. A transparent glass platform (10 cm) is submerged in the centre of one of the quadrants, denoted as the target quadrant, by approximately one centimetre. Four different high contrast distal cues were placed on the walls surrounding the basin. The water temperature was kept around 24–25 degree Celsius and the light intensity was approximately 220 lx in the centre of the pool.

Each trial started with a mouse being placed on the platform for 30 s. Mice were then placed in the water facing the wall pseudo-randomly at one of the six start positions of the basin and allowed to search for the platform or until 60 s had elapsed. At the completion of each training trial, the mice were allowed to remain on the platform for 30 s. Mice were given 3 trials per day until reaching a criterion threshold of learning by escaping the water in less than 30 s. After criterion was reached, a probe trial was conducted in which the platform was removed. Mice were placed facing the wall in the quadrant directly opposite the previous location of the hidden platform and allowed to search for 60 s. The day after the probe trial, the mice were given the visually cued test, where the platform location was changed but was marked with a distinct local cue. The distant visual cues were hidden from sight of the mice and visual acuity, swimming ability and motivation for escape were assessed by how well the mice could escape the pool using only the local cue. Three trials were given of the visual cued water maze. Data analysis was carried out by the automated video tracking TSE VideoMot software. If the mouse did not explore the maze but floated upon placement in the water, the data were excluded from the analysis. Mice were also excluded based on standard exclusion criteria: excessive thigmotaxis, obvious visual impairment, excessive corkscrew swimming pattern, and obvious sensorimotor dysfunction. Genotype and sex did not affect likelihood of exclusion. Nine of the twenty Syn1Cre male mice displayed excessive floating, but these mice were not removed as that was a phenotype of the Syn1Cre male mice.

### Molecular biology

#### Quantitative real time PCR

For the analysis of DNA expression, dissected organs were immediately transferred to a reagent tube and frozen in liquid nitrogen. Tissue was stored at − 80 °C until use. RNA was extracted according to the manufacturer’s instructions using the TRIzol™ Reagent (ThermoFisher, 15596018) in Lysing Matrix D tubes (speed 6 for 40 s) (MP Biomedicals, 6913-500). RNA was precipitated with the aid of GlycoBlue Coprecipitant (ThermoFisher, AM9515) overnight at − 80 °C. Isolated RNA was treated with DNase using the DNA-free™ kit (ThermoFisher, AM1906) to remove any contaminating DNA according to manufacturer’s instructions. Finally, cDNA was prepared with the SuperScript® III First-Strand Synthesis SuperMix (ThermoFisher, 18080400) for qPCR. Samples of cDNA mixed with the PowerUp SYBR Green Master Mix (ThermoFisher, 4368706) and primers validated using a standard curve, were loaded in technical quadruplicates for qPCR on a QuantStudio™ 6 Flex Real-Time PCR System (ThermoFisher, 4485691). The ΔΔCT method was used to provide gene expression values after normalising to the known reference gene B2M. Primer sequences used for q-RT-PCR are shown in Supplementary Table S2.

### Bone size measurement

Mouse femurs were removed from the animal and snap frozen at − 80 °C. Frozen femurs were thawed at 4 °C before scanning. Femurs were scanned with a high resolution µCT scanner (SkyScan 1176, Bruker, Belgium) with an isotropic voxel size of 8.8 µm^3^. The x-ray settings for each scan were 50 kV and 200 µA using a 0.5 mm aluminium filter. All scans were performed over 360 degrees with a rotation step of 0.3 degrees and a frame averaging of one. Images were reconstructed and analysed using NRecon and DataViewer software, respectively (Bruker, Belgium).

### Mouse metabolic phenotyping

A different cohort of mice was bred for metabolic phenotyping to ensure there are no confounding effects from previous experiments. Metabolic phenotyping was conducted in dedicated testing rooms depending on the assay starting at 08:00–09:00 during the light phase of the light/dark cycle. Order of the phenotyping pipeline was performed from the least to most stressful assay and mice were given at least a week of rest in between assays (Supplementary Fig. S4). Mice were three months old at the start of the phenotyping pipeline. Mice were randomised and experimenters were blinded to genotype and all experiments were performed by a male and female scientist.

#### Body composition

Body fat and lean mass content were measured in vivo by nuclear magnetic resonance using a minispec LF50H (Bruker Corporation).

#### Collection of blood samples and determination of blood glucose levels

Mice were assessed after an overnight fast and three hours after light cycle onset. Blood glucose levels were determined by collecting a small drop of blood from the tail of mice and placing it on an automatic glucose monitor (Accu-Check Aviva, Roche). Determination of blood glucose was always performed in the morning to avoid deviations due to circadian variations.

#### Insulin tolerance test

Mice were assessed without fasting three hours after light cycle onset. After determination of basal blood glucose levels, each animal received an intraperitoneal injection of insulin (0.75 U/kg body weight) (Sanofi). Blood glucose levels were measured 15, 30 and 60 min after insulin injection.

#### Glucose tolerance test

Glucose tolerance tests were performed in the morning with animals after a 16 h fast. After determination of fasted blood glucose levels, each animal received an intraperitoneal injection of 20% (w/v) glucose (10 ml/kg body weight). Blood glucose levels were measured 15, 30, 60 and 120 min after the glucose injection.

#### Indirect calorimetry

Indirect calorimetry, locomotor activity, drinking and feeding were monitored for singly housed mice in purpose-built cages (Phenomaster, TSE Systems). Parameters such as food consumption, respiration, and locomotor activity were measured continuously for 48 h after one day of acclimatisation and two days of training in similar cages. Values for locomotor activity were averaged for active and inactive phases separately for the 48-h duration with the exception of the first and last hour of each phase due to increased variability. Alternatively, for locomotion the average of the two days was calculated and plotted with standard error of the mean error bars. Metabolic rate was assessed by regression analysis using body weight as a covariate as recommended^33,34^.

### Statistics

Two-group comparisons were made using two-tailed, unpaired Student’s t-test unless otherwise stated. However, if data was not normally distributed then Mann Whitney U test was performed. For comparisons of two factors (for example, phase * genotype or sex*genotype), two-way ANOVA was used, followed by Sidak’s post-test if interaction between main effects was significant.

Numbers of mice were estimated to be sufficient to detect statistically meaningful differences of at least 20% between or among groups using standard power calculations with α = 0.05 and power of 0.8 on the basis of similar experiments conducted in our group. Homogeneity of variance and normality of residuals were assessed, and appropriate corrections were made if necessary. All experiments were performed in a randomised and blinded fashion. Data were analysed statistically using GraphPad Prism 9.0, outliers were removed from analysis based on a Grubb’s test. The value of α was 0.05, and data are expressed as *P < 0.05; **P < 0.01; ***P < 0.001; ****P < 0.0001. Number of animals reported at the bottom of the bars for each condition. All error bars correspond to standard deviation except for longitudinal glucose and insulin sensitivity as well as rotarod experiments where standard error of the mean is reported. ANCOVA analyses were plotted with 95% confidence interval bands. Detailed P values for non-significant comparisons, test statistic values, and degrees of freedom are included in Supplementary Table S1.

## Supplementary Information

Supplementary Figures.

Supplementary Table S1.

Supplementary Table S2.

## Data Availability

Datasets are available upon request from the corresponding authors—Maarouf Baghdadi and Linda Partridge. The raw data supporting the conclusions of this article will be made available by the authors, without undue reservation, to any qualified researcher.
