# Supplementary material for: Sex-specific effects of Cre expression in Syn1Cre mice
Source: Sci Rep. 2023 Jun 20;13:10037. doi: 10.1038/s41598-023-37029-9 (PMC10281999; doi:10.1038/s41598-023-37029-9)
Supplement: Supplementary file 1 — Supplementary Figures. [file 41598_2023_37029_MOESM1_ESM.docx]

**Sex-specific effects of Cre expression on behaviour in Syn1Cre mice**

**Maarouf Baghdadi ¹, Andrea Mesaros ¹, Martin Purrio ¹, Linda Partridge ¹ ²**

1. Max-Planck Institute for Biology of Ageing, Cologne, Germany

2. Institute of Healthy Ageing, and GEE, UCL, London, UK

Corresponding authors: Maarouf Baghdadi ([mbaghdadi@age.mpg.de](mailto:mbaghdadi@age.mpg.de)) and Linda Partridge ([Partridge@age.mpg.de](mailto:Partridge@age.mpg.de))

**Supplementary material:**

Supplementary Figure S1 – S3

## Supplementary Figure S1: Additional outcome measures from memory tasks

**(a)** Total swim path during the 60 second probe trial revealed significant reduction in Syn1Cre mice. **(b)** Total swim path in the cued variation of the water maze showed no significant change in Syn1Cre mice. **(c)** Assessment of swim speed during the probe trial revealed significant reduction in Syn1Cre mice. **(d)** Swim speed in the cued water maze variant revealed significant reduction in Syn1Cre mice. **(e)** Measurement of total distance covered in the Y-maze was unaltered in Syn1Cre mice. **(f)** Locomotion speed in the Y-maze was not affected in Syn1Cre mice. Number of animals reported at the bottom of the bars. All error bars correspond to standard deviation of the mean. Asterisks denote the following: *P < 0.05, **P < 0.01, ***P < 0.001, ****P < 0.0001. For detailed statistical values see Supplementary Table S1.

## Supplementary Figure S2: Ct values for Cre and Cre-hGH in the hypothalamus of Syn1Cre mice

**(a)** Measuring level of Cre transcripts in the hypothalamus of Syn1Cre mice revealed a genotype specific expression of Cre recombinase (some samples did not reach detection threshold). **(b)** Measurement of Cre-hGH transcripts in the hypothalamus of Syn1Cre mice detected expression only in Syn1Cre mice and not wild type littermates (some samples did not reach detection threshold). Asterisks denote the following: *P < 0.05, **P < 0.01, ***P < 0.001, ****P < 0.0001. For detailed statistical values see Supplementary Table S1.

## Supplementary Figure S3: Additional metabolic measures of Syn1Cre mice

**(a)** Assessment of fat mass as a percentage of body weight did not reveal any difference in body composition of Syn1Cre mice. ANCOVA on night-time energy expenditure of Syn1Cre mice showed no significant difference between male **(b)** or female **(c)** Syn1Cre and their respective wild type littermates. Respiratory exchange ratio of male **(d)** and female **(e)** Syn1Cre mice did show any significant difference due to genotype compared to wild type littermates. Number of animals reported at the bottom of the bars. All error bars correspond to standard deviation of the mean. For ANCOVA analysis the 95% confidence interval is plotted. Asterisks denote the following: *P < 0.05, **P < 0.01, ***P < 0.001, ****P < 0.0001. For detailed statistical values see Supplementary Table S1.

##
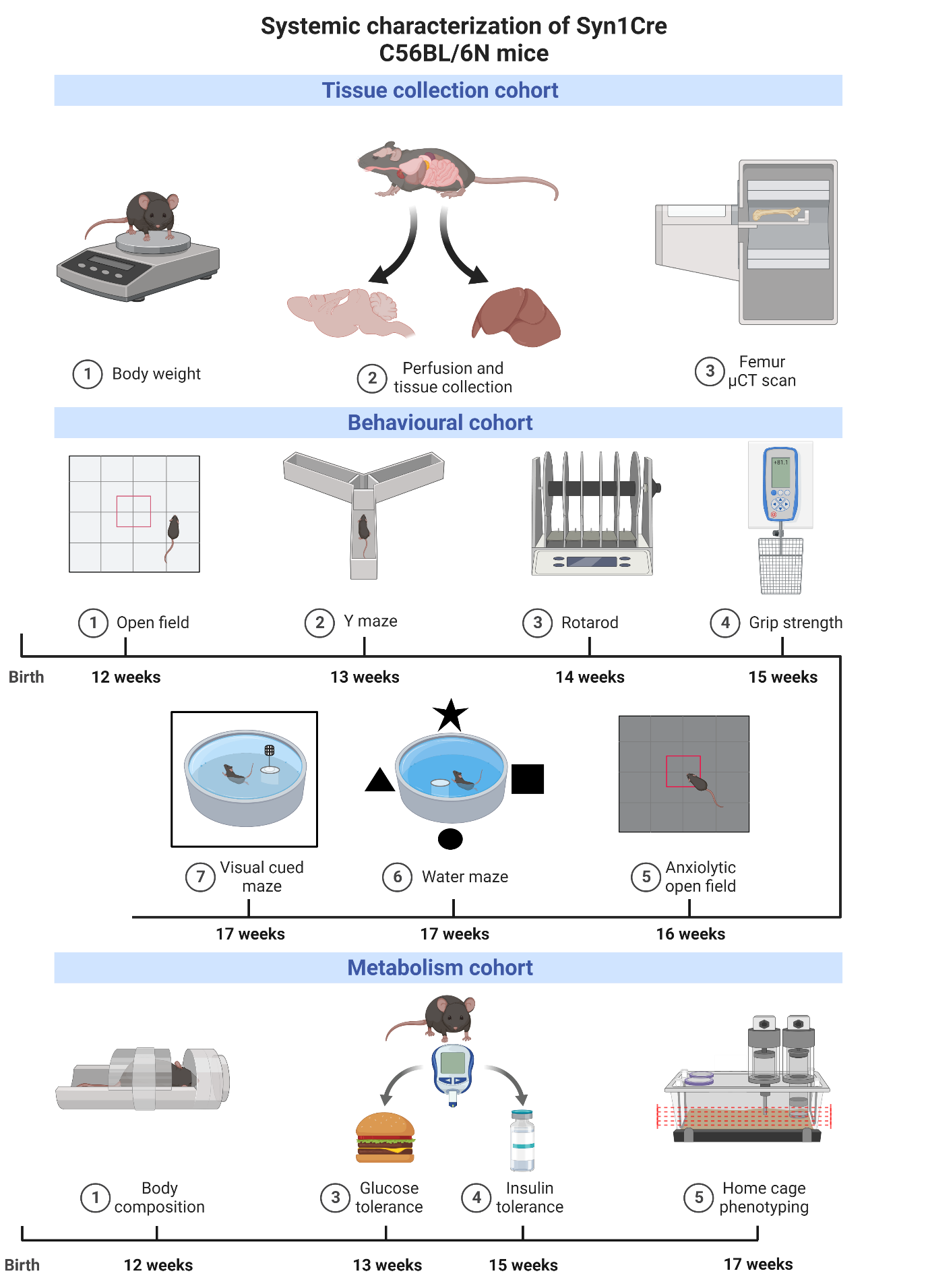
Supplementary Figure S4: Illustration for phenotyping pipeline Syn1Cre mice

A schematic of the three independent mouse cohorts generated to assess molecular, behavioural and metabolic characteristics of male and female Syn1Cre mice.
